# Effects of preoperative physiotherapy on signs and symptoms of pulmonary collapse and infection after major abdominal surgery: secondary analysis of the LIPPSMAck-POP multicentre randomised controlled trial

**DOI:** 10.1186/s13741-021-00206-3

**Published:** 2021-10-25

**Authors:** I. Boden, J. Reeve, I. K. Robertson, L. Browning, E. H. Skinner, L. Anderson, C. Hill, D. Story, L. Denehy

**Affiliations:** 1 Department of Physiotherapy, Launceston General Hospital, Launceston, Australia; 2 Melbourne School of Health Sciences, The University of Melbourne, Melbourne, Australia; 3 School of Clinical Sciences, Faculty of Health and Environmental Sciences, Auckland University of Technology, Auckland, New Zealand; 4 Physiotherapy Department, North Shore Hospital, Waitemata District Health Board, Auckland, New Zealand; 5 School of Health Sciences, University of Tasmania, Launceston, Australia; 6 Clifford Craig Foundation, Launceston General Hospital, Launceston, Australia; 7 Directorate of Community Integration, Allied Health and Service Planning, Western Health, Melbourne, Australia; 8 Faculty of Medicine Nursing and Health Science, Monash University, Frankston, Australia; 9 Department of Medicine, The Alfred Hospital, Melbourne, Australia; 10 Physiotherapy Department, North West Regional Hospital, Burnie, Australia; 11 Anaesthesia Perioperative and Pain Medicine Unit, The University of Melbourne, Melbourne, Australia; 12 Melbourne Clinical and Translational Science Research Platform, Melbourne, Australia; 13 Allied Health Research, Peter McCallum Cancer Centre, Melbourne, Australia

**Keywords:** Pulmonary complications, Preoperative, Physiotherapy, Antibiotics, Oxygen therapy, Abdominal surgery, Breathing exercises

## Abstract

**Background:**

Preoperative education and breathing exercise training by a physiotherapist minimises pulmonary complications after abdominal surgery. Effects on specific clinical outcomes such as antibiotic prescriptions, chest imaging, sputum cultures, oxygen requirements, and diagnostic coding are unknown.

**Methods:**

This post hoc analysis of prospectively collected data within a double-blinded, multicentre, randomised controlled trial involving 432 participants having major abdominal surgery explored effects of preoperative education and breathing exercise training with a physiotherapist on postoperative antibiotic prescriptions, hypoxemia, sputum cultures, chest imaging, auscultation, leukocytosis, pyrexia, oxygen therapy, and diagnostic coding, compared to a control group who received a booklet alone. All participants received standardised postoperative early ambulation. Outcomes were assessed daily for 14 postoperative days. Analyses were intention-to-treat using adjusted generalised multivariate linear regression.

**Results:**

Preoperative physiotherapy was associated with fewer antibiotic prescriptions specific for a respiratory infection (RR 0.52; 95% CI 0.31 to 0.85, *p* = 0.01), less purulent sputum on the third and fourth postoperative days (RR 0.50; 95% CI 0.34 to 0.73, *p* = 0.01), fewer positive sputum cultures from the third to fifth postoperative day (RR 0.17; 95% CI 0.04 to 0.77, *p* = 0.01), and less oxygen therapy requirements (RR 0.49; 95% CI 0.31 to 0.78, *p* = 0.002). Treatment effects were specific to respiratory clinical coding domains.

**Conclusions:**

Preoperative physiotherapy prevents postoperative pulmonary complications and is associated with the minimisation of signs and symptoms of pulmonary collapse/consolidation and airway infection and specifically results in reduced oxygen therapy requirements and antibiotic prescriptions.

**Trial registration:**

ANZCTR 12613000664741; 19/06/2013.

**Supplementary Information:**

The online version contains supplementary material available at 10.1186/s13741-021-00206-3.

## Background

Pathophysiological effects of anaesthesia and the abdominal incision during major abdominal surgery cause deleterious effects on lung volumes, mucociliary clearance, and cough strength (Miskovic and Lumb 2017). Atelectasis is almost inevitable in the immediate postoperative period, with up to 90% of patients having under-aerated lung tissue occupying up to a quarter of lung fields in the first hour after surgery (Lundquist et al. 1995), despite advances in perioperative surgical and anaesthetic practices (Pereira et al. 2018). Approximately 50% of patients continue to have notable atelectasis 24 h after surgery (Touw et al. 2019). Unresolved atelectasis is considered a primary pathogenic precursor for microbial contamination (van Kaam et al. 2004), pneumonia, and acute respiratory distress syndrome (Tusman et al. 2012). Postoperative pulmonary complications (PPC) are common after upper abdominal surgery and have considerable impacts on morbidity, mortality, and hospital costs (Miskovic and Lumb 2017; Fleisher and Linde-Zwirble 2014; Abbott et al. 2018; Boden et al. 2018a).

The Lung Infection Prevention Post Surgery Major Abdominal with Pre-Operative Physiotherapy (LIPPSMAck-POP) study was a phase-three, binational, multicentre, randomised placebo-controlled trial (Boden et al. 2018a) that found preoperative physiotherapy independently halves PPC rates, including pneumonia, after major abdominal surgery. The primary focus of the intervention was to educate, enable, and motivate participants to perform hourly sets of deep breathing and coughing exercises immediately upon waking from surgery. The hypothesis was that repetitive independent performance of breathing exercises in the early postoperative period would reverse atelectasis and improve sputum clearance resulting in reduced risk of a PPC. In a deliberate effort to minimise Hawthorne effects, the performance of breathing exercises was not directly measured (Boden et al. 2018a; Boden et al. 2015; Boden et al. 2018b). Consequently, it cannot be stated with absolute certainty that the mechanism of effect for PPC reduction in the LIPPSMAck-POP trial were self-directed breathing exercises. Additionally, the primary outcome was a PPC identified using a diagnostic screening tool, the Melbourne Group Score (Abbott et al. 2018; Boden et al. 2018a; Boden et al. 2015). There is some debate surrounding the validity of generic PPC diagnosis tools and their relationship with clinical outcomes (Abbott et al. 2018). Specific effects on chest imaging, sputum cultures, antibiotic prescriptions, oxygen requirements, and administrative coding from the LIPPSMAck-POP trial have not been reported. Positive effects on physiological outcomes would provide concurrent validity to the primary results and support the hypothesis that preoperative physiotherapy enables patients to perform breathing exercises after surgery. This information would also assist perioperative health professionals and hospital administrators to understand the impact that physiotherapy has on important tangible postoperative clinical outcomes, and this may strengthen the case for widespread implementation of this service.

## Methods

The first aim of these exploratory secondary analyses was to investigate the distribution and occurrence of common postoperative clinical signs and symptoms related to respiratory pathology using a priori prospectively collected data and acquired administrative clinical coding. The second aim was to test the following two hypotheses: (1) preoperative physiotherapy that reduces PPCs as assessed using the Melbourne Group Score should also affect clinical outcomes specifically related to pulmonary collapse and infection, such as oxygen therapy usage and antibiotic prescriptions; yet should not affect complications physiologically unlikely to be prevented with breathing exercises such as pulmonary emboli (Boden et al. 2018a) or non-respiratory-related infections; and (2) if self-directed breathing exercises are the primary method of effect, benefits in patients sedated and mechanically ventilated after surgery should not be expected due to their inability to perform breathing exercises. LIPPSMAck-POP was a multicentre, parallel-group, double-blinded (assessors and patients), randomised controlled trial conducted at three hospitals in Australia and New Zealand. It tested the effectiveness of a single preoperative respiratory education and coaching session delivered by a physiotherapist, compared to an information booklet alone, to reduce PPCs following major abdominal surgery (Boden et al. 2018a). LIPPSMAck-POP was prospectively registered (ANZCTR 12613000664741), approved by local ethics committees, with participants providing written informed consent. Design, methodology, primary results, qualitative findings, and health economic outcomes are published in detail elsewhere (Boden et al. 2018a; Boden et al. 2015; Boden et al. 2018b; Boden et al. 2020).

As reported previously, 432 adults attending an outpatient pre-admission clinic within 6 weeks of elective major abdominal surgery were block randomised without stratification via concealed allocation to receive preoperative physiotherapy (intervention, *n* = 218) or an information booklet (control, *n* = 214). Intervention participants were provided with the booklet and a single 30-min education session about the effect of anaesthesia and abdominal surgery on mucociliary clearance, lung volumes, and the consequences of bacterial stagnation in the lungs. Patients were educated that self-directed breathing exercises were vital in preventing pneumonia and were directed to commence these exercises immediately after surgery. Participants were coached in the breathing exercises and provided with memory cues to prompt hourly postoperative performance. Control participants received a placebo information booklet. Following surgery, all participants received standardised postoperative ambulation, and no additional prophylactic chest physiotherapy or incentive spirometers were provided.

The median age of participants was 65 (range 52–75) and proportionally more were males (61%). Surgery was mainly of curative intent for cancer (69%), requiring major colorectal (49%), renal (26%), or upper gastrointestinal/hepatobiliary procedures (24%). Operations were generally longer than 2 h (64%) via open upper midline (49%) or subcostal (18%) incisions (Boden et al. 2018a).

All data utilised for these secondary analyses were prespecified a priori, prospectively registered, and collected prospectively by masked assessors. Patients, postoperative physiotherapists, nurses, surgeons, anaesthetists, and clinical coders were unaware of group allocation. Apart from the clinical coding analysis, these post hoc exploratory secondary analyses were not pre-specified within the original trial registration. During trial design, the primary intent was a comparative analysis on the primary outcome of PPC minimisation and key secondary outcomes of hospital utilisation, costs, and patient-reported health-related quality of life. Secondary analyses exploring the comparative effects to specific postoperative clinical criteria and sub-group effects in those mechanically ventilated or not, were conceptualised only once the LIPPSMAck-POP results were analysed and questions surrounding trial validity and clinical applicability were raised.

Participants were assessed daily for a PPC using a modified Melbourne Group Score (Boden et al. 2018a; Boden et al. 2015) over the first 14 hospital days. This was the LIPPSMAck-POP trial’s primary outcome. The Melbourne Group Score consists of eight clinical criteria; abnormal chest auscultation, abnormal sputum colour, hypoxemia on room air, pyrexia, collapse/consolidation on chest x-ray (CXR) or computerised tomography (CT), leukocytosis, infected sputum culture, and a medical diagnosis of a pulmonary complication or antibiotic prescription specific for a pulmonary infection (see Additional file 1, Box 1S and Table 1S for detailed description and standardised collection rules). The concurrent presence of four or more of these criteria in a calendar day triggered a PPC diagnosis.

For the purposes of these secondary analyses, each criterion within the Melbourne Group Score was considered separately for between-group differences. Prescription of antibiotics specific for a respiratory infection was determined by probing medication charts for antibiotics prescribed in direct response to respiratory deterioration documented in the medical record by a physician. The medical team was contacted for clarification if required. The first day of antibiotic initiation was recorded. Auscultation abnormalities, sputum colour abnormalities, peripheral oxyhaemoglobin (SpO_2_) desaturation < 90% on room air, and temperature over 38 °C were assessed daily and purposively for this trial. White blood cell counts, sputum sampling, and chest imaging were not ordered purposively and daily for this trial rather the collection of these criteria was based on pragmatic clinical practice as required by masked surgical or anaesthesia teams. Collapse/consolidation on CXR and CT, and abnormal pathology tests were reported by independent blinded radiologists or pathologists, and these results were extracted from hospital databases by the trial’s assessors. A physician diagnosis of a PPC was specific documentation of pneumonia, upper or lower respiratory tract infection, or atelectasis written by a doctor in the medical record.

Mode of oxygen therapy and mechanical ventilation was collected daily until the 14th hospital day. At participating hospitals, supplemental oxygen therapy was mandatory during administration of opioid-based intravenous analgesia. For the purposes of this study, this was not counted as oxygen therapy required to manage a respiratory deterioration (Abbott et al. 2018) if the oxygen therapy was delivered in the absence of signs or symptoms of respiratory dysfunction (hypoxemia, chest imaging abnormalities, or physician documentation) and only for the purposes of the administration of intravenous analgesia.

As per standard hospital administrative practices, masked clinical coders assessed the medical record and classified each participant’s episode of care for postoperative complications according to the World Health Organisation (WHO) International Classification of Diseases (ICD-10) coding set (International Classification of Diseases (ICD-10) [World Health Organisation web site] 2018). Diagnostic coding is used to assist governmental administrative reporting and for case-mix calculated activity-based funding of hospital services. Clinical coding was extracted and collated by masked trial assessors from hospital databases. Codes were grouped into intraoperative complications, non-respiratory postoperative complications, and respiratory postoperative complications. Respiratory complication coding was further divided into those related to atelectasis and airway infection, such as pneumonia, and those not related, such as a pneumothorax (Abbott et al. 2018). See Additional file 1, Table 2S, for full listing of relevant codes, diagnostic labels, and cohorting rules.

The time from end of anaesthesia to extubation from mechanical ventilation was collected a priori (Boden et al. 2015). For this secondary analysis, participants were separated into two cohorts for sub-group analysis of the treatment effect to the primary PPC endpoint; those sedated and continuously mechanically ventilated immediately after surgery, and those participants extubated on cessation of anaesthesia.

An a priori power calculation was not performed for these exploratory secondary analyses. Categorical values are presented as counts and percentages. Event rates for oxygen usage and mechanical ventilation are a single positive occurrence anytime within the first 14 postoperative days. Clinical signs and symptoms are reported two ways. Firstly, the proportion of participants who had a single positive occurrence anytime within the first 14 postoperative days, and secondly, a daily event rate where the daily proportion of participants with a positive incidence on each of the first seven postoperative days is represented graphically with 95% confidence intervals. Clinical criteria event rates were compared using adjusted generalised linear Poisson modelling. Diagnostic coding total event rates for each coding category were compared using adjusted multivariate robust random effects binary logistic generalised linear regression. The mean difference in total number of respiratory specific codes was assessed using adjusted multivariate linear regression. PPC incidence according to postoperative ventilation status and differences in antibiotic prescriptions was analysed using survival-time regression analysis and graphically illustrated using Kaplan-Meier methods. All data are intention-to-treat, relative risk, or mean difference between groups with 95% confidence intervals and two-tailed *p* values, adjusted for known baseline imbalances between groups in age, respiratory comorbidity, and surgical category (Boden et al. 2018a). Detailed description of the statistical analysis plan, covariates, and adjustment modelling are available open access (Boden et al. 2018a; Boden et al. 2015). Analyses were performed using SPSS (V23, IBM) and STATA (V14.1, Stata Corp.).

## Results

From 2013 to 2015, 441 participants (intervention, *n* = 218; control, *n* = 214; withdrawn, *n* = 9) were recruited in this double-blinded, multicentre randomised controlled trial across two countries. Baseline, clinical characteristics, and flow through trial are previously published (Boden et al. 2018a). An auscultation abnormality was the most common positive finding on postoperative day 1 (58% of all participants; see Additional file 1, Figure 1S), followed by leukocytosis (52%), hypoxemia on room air (21%), abnormal sputum colour (14%), and chest imaging findings of collapse/consolidation (10%). Daily rates of fever and positive sputum cultures were < 5%. Auscultation abnormalities, leukocytosis, and hypoxemia became less prevalent over time. The rate of change in daily purulent sputum production was notably different, elevating from 14% of all participants to 22% over the first 3 days.

**Fig. 1:**
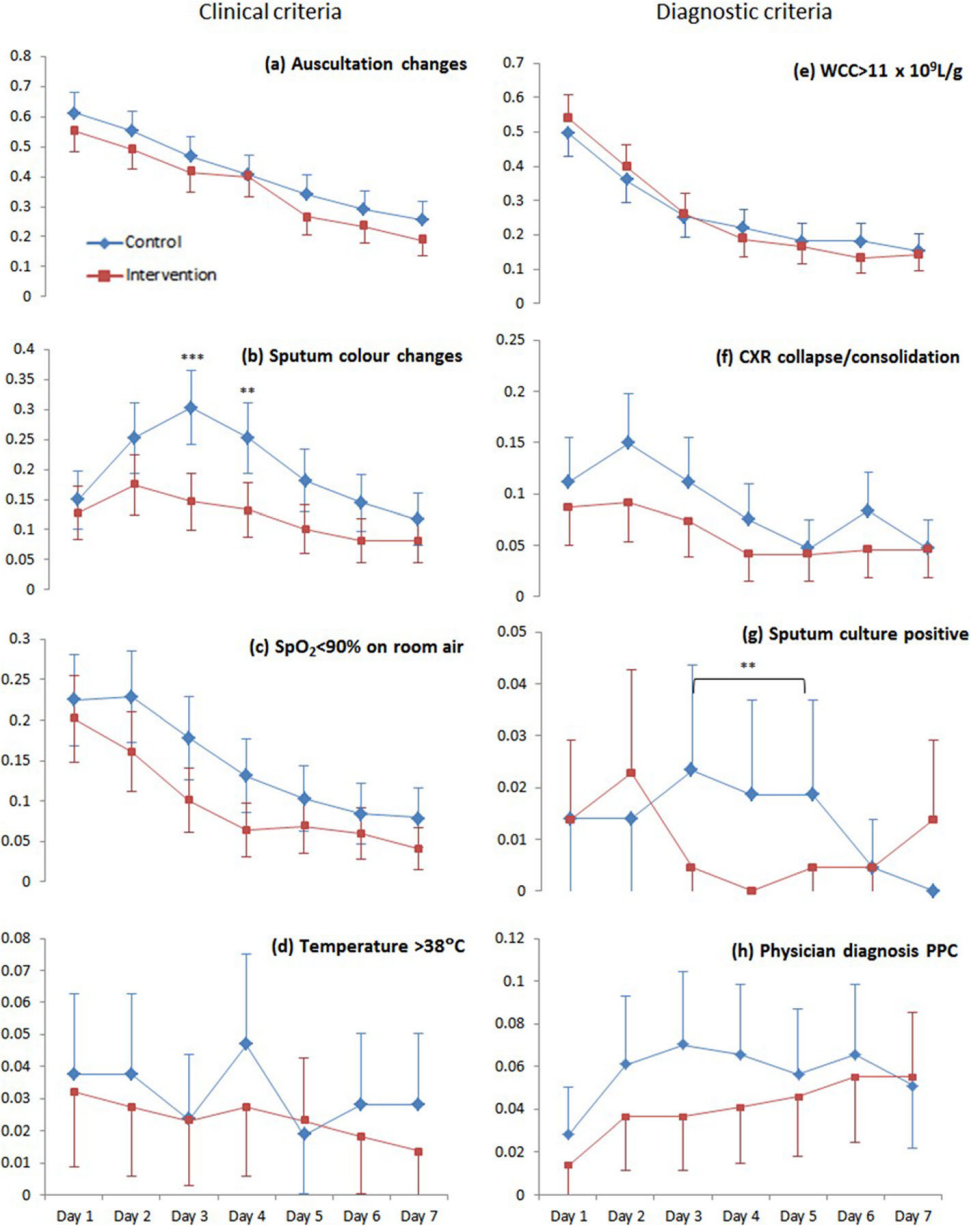
Daily rates of clinical criteria. Data are proportions with error bars showing 95% confidence intervals. Intervention (red lines); control (blue lines). *p* values: *p = 0.02; **p = 0.01; ***p < .001

Between the two groups (Fig. 1), the intervention group was associated with approximately half the risk of purulent sputum being detected on the third day (RR 0.54, 95% CI 0.33 to 0.90; *p* = 0.017; Fig. 1b) and had an estimated 80% less risk of a positive sputum culture result from the third to the fifth days (RR 0.12, 95% CI 0.02 to 0.72; *p* = 0.02; Fig. 1g) compared to control participants. Although a separation between groups favouring the intervention group is graphically evident in other daily criteria, statistical significance was not found. These criteria had low daily event rates (pyrexia and physician diagnosis; Fig. 1d, h) and small effect sizes (auscultation changes and hypoxemia on room air; Fig. 1a, c), limiting the statistical power for these secondary endpoints.

**Fig. 2:**
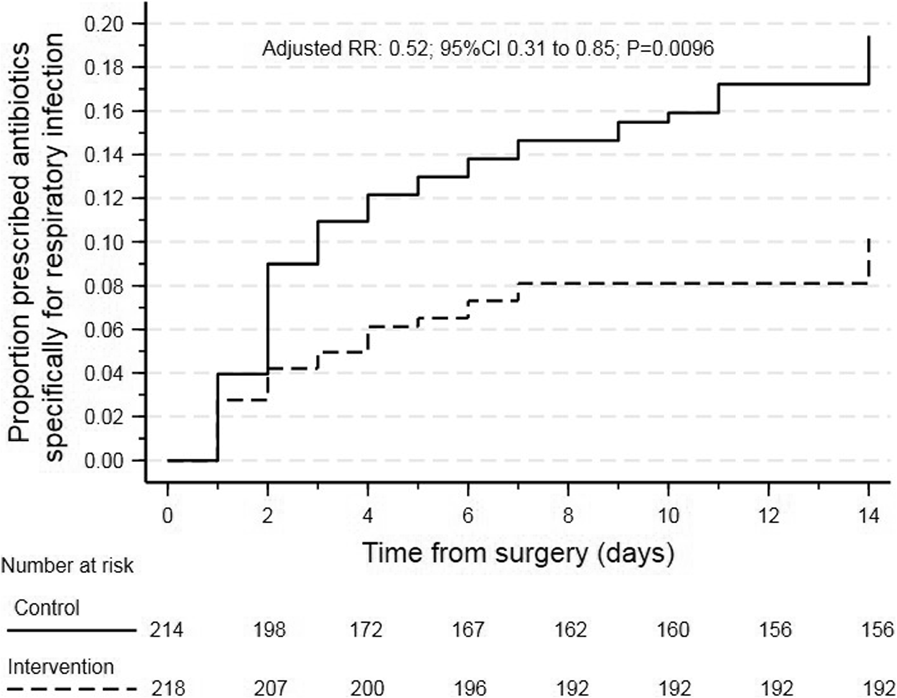
Cumulative rate of antibiotic prescriptions specific for a respiratory infection. Data are proportions. Intervention (dotted line); control (solid line)

When considering the proportional occurrence of criteria at any time in the first 14 postoperative days (Table 1), the intervention was associated with an estimated 40% less risk of being prescribed antibiotics specific for a respiratory infection (RR 0.60, 95% CI 0.40 to 0.91, *p* = 0.016; Table 1) compared to the control group. Differences in antibiotic prescriptions rates were evident from the second day (Fig. 2). No statistically significant treatment effects between groups were detected in the event rate of other criteria occurring at least once anytime over the first 14 days (Table 1).

**Table 1:** Treatment effects to postoperative clinical criteria and oxygen therapy requirements. Values are number (proportion)

	Control (*n* = 214)	Intervention (*n* = 218)	Adjusted RR (95% CI)	*p* value
**Clinical criteria 14-day event rates**				
Auscultation abnormal	161 (75%)	151 (69%)	0.94 (0.84 to 1.06)	0.35
Sputum colour abnormal	90 (42%)	81 (37%)	0.94 (0.74 to 1.18)	0.57
Hypoxemia	74 (35%)	64 (29%)	0.93 (0.71 to 1.23)	0.63
Pyrexia	27 (13%)	28 (13%)	1.04 (0.63 to 1.73)	0.87
Collapse/consolidation on CXR/CT	57 (27%)	39 (18%)	0.74 (0.52 to 1.05)	0.09
Leukocytosis	134 (63%)	141 (65%)	1.05 (0.92 to 1.22)	0.43
Sputum culture positive for infection	15 (7.0%)	9 (4.1%)	0.67 (0.30 to 1.48)	0.33
Physician diagnosis of PPC in medical record	37 (17%)	28 (13%)	0.86 (0.51 to 1.35)	0.51
Respiratory antibiotics prescribed	53 (25%)	29 (13%)	0.60 (0.40 to 0.91)	0.02
**Oxygen therapy and mechanical ventilation**				
No oxygen therapy	129 (60%)	172 (79%)	0.49 (0.31 to 0.78)	0.002
Standard oxygen therapy	48 (22%)	24 (11%)		
High flow oxygen therapy	25 (12%)	10 (4.6%)		
Invasive or non-invasive mechanical ventilation	12 (5.6%)	12 (5.5%)		

*Abbreviations*: *RR* relative risk, *CI* confidence interval, *PPC* postoperative pulmonary complications, *CXR* chest X-ray, *CT* computerised tomography

Data are event rates within the first 14 postoperative hospital days compared using mixed effects general linear Poisson regression reported as relative risk with 95% CI, adjusted for age, respiratory comorbidity, and surgical category, with exposure time as the time to cessation of observations

Oxygen therapy was classified as the most intensive therapy provided to a patient, a rank-order scale, with comparison estimated as odds ratio using ordered linear regression adjusted as above

Intervention participants were at less risk of requiring oxygen therapy during the first 14 postoperative days (RR 0.49, 95% CI 0.31 to 0.78, *p* = 0.002; Table 1) with half as many intervention participants requiring standard supplemental or high-flow oxygen therapy. No statistical difference was detected for non-invasive or invasive mechanical ventilation requirements with a low event rate of 5% per group.

Intervention participants had fewer counts overall of diagnostic coding for pneumonia, pulmonary collapse, acute respiratory failure, and other respiratory diagnostic codes related to atelectasis and airway infection when compared to control participants (Table 2). A difference between groups was not detected in the administrative diagnostic coding of respiratory complications unrelated to atelectasis or airway infection (pleural effusion, pneumothorax, pulmonary emboli), nor in the incidence of intraoperative or non-respiratory postoperative complications (Table 2).

**Table 2:** Clinical coding of postoperative complications. Values are number (proportion) or mean (SD)

	Control *n* = 214	Intervention *n* = 218	Adjusted RR or mean difference (95% CI)	*p* value
**Clinical coding (ICD-10) event rates of intraoperative and non-respiratory postoperative complications**				
Intraoperative complications				
Laceration	12 (5.6%)	22 (10%)	1.71 (0.84 to 3.50)	0.14
Haemorrhage	13 (6.1%)	16 (7.3%)	1.26 (0.61 to 2.62)	0.53
Postoperative complications—surgical				
Wound infection	25 (12%)	22 (10%)	0.90 (0.52 to 1.55)	0.70
Wound dehiscence	7 (3.3%)	6 (2.8%)	0.92 (0.29 to 2.89)	0.89
Anastomosis leak	1 (0.5%)	3 (1.4%)	3.16 (0.26 to 37.5)	0.36
Postoperative complications—general				
Paralytic ileus	42 (20%)	36 (17%)	0.87 (0.58 to 1.31)	0.50
Hypovolemia	26 (12%)	26 (12%)	1.04 (0.62 to 1.75)	0.87
Delirium/altered conscious state	25 (12%)	21 (9.6%)	1.01 (0.60 to 1.71)	0.97
Other infections	24 (11%)	18 (8.5%)	0.84 (0.47 to 1.51)	0.56
Urinary tract infection	18 (8.4%)	12 (5.5%)	0.85 (0.44 to 1.64)	0.63
Sepsis	13 (6.1%)	7 (3.2%)	0.63 (0.26 to 1.57)	0.33
Acute kidney injury	10 (4.7%)	8 (3.7%)	0.85 (0.33 to 2.20)	0.74
Pressure ulcer	12 (5.6%)	3 (1.4%)	0.31 (0.09 to 1.09)	0.07
Cardiac	7 (3.3%)	10 (4.6%)	1.72 (0.65 to 4.58)	0.28
Hypervolemia	4 (1.9%)	7 (3.2%)	1.96 (0.57 to 6.71)	0.28
Deep vein thrombosis	2 (0.9%)	3 (1.4%)	1.69 (0.30 to 9.60)	0.56
**Clinical coding (ICD-10) event rates of respiratory postoperative complications**				
Coding of respiratory complications hypothesised to be preventable with breathing exercises				
Nonspecific pulmonary problem^a^	26 (12%)	11 (5.0%)	0.48 (0.24 to 0.95)	0.03
Pulmonary collapse	21 (9.8%)	16 (7.3%)	0.83 (0.45 to 1.54)	0.56
Pneumonia	19 (8.9%)	15 (6.9%)	0.94 (0.49 to 1.79)	0.84
Acute respiratory failure	11 (5.1%)	5 (2.3%)	0.54 (0.19 to 1.49)	0.23
Mean number of codes per participant	0.36 (0.48)	0.22 (0.41)	0.71 (0.41 to 1.22)	0.22
Coding of respiratory complications unlikely to be preventable with breathing exercises				
Pleural effusion	12 (5.6%)	8 (3.7%)	0.75 (0.31 to 1.76)	0.50
Pneumothorax	8 (3.7%)	12 (5.5%)	1.42 (0.59 to 3.43)	0.43
Nonspecific pulmonary problem^b^	6 (2.8%)	11 (5.0%)	1.74 (0.64 to 4.70)	0.28
Pulmonary emboli	2 (0.9%)	4 (1.4%)	2.57 (0.47 to 14.2)	0.28
Mean number of codes per participant	0.13 (0.34)	0.16 (0.37)	1.30 (0.71 to 2.36)	0.40

*Abbreviations*: *RR* relative risk, *CI* confidence interval, *ICD-10* International Classification of Diseases Version 10

Data compared using relative risk or mean difference with 95% CI adjusted for age, respiratory comorbidity, and surgical category

^a^non-specific coding of a pulmonary problem/symptom in conjunction with coding specific to pulmonary collapse or infection (see Additional file 1, Table 2S)

^b^Non-specific coding of a pulmonary problem/symptom in absence of coding specific to pulmonary collapse or infection (see Additional file 1, Table 2S)

**Fig. 3:**
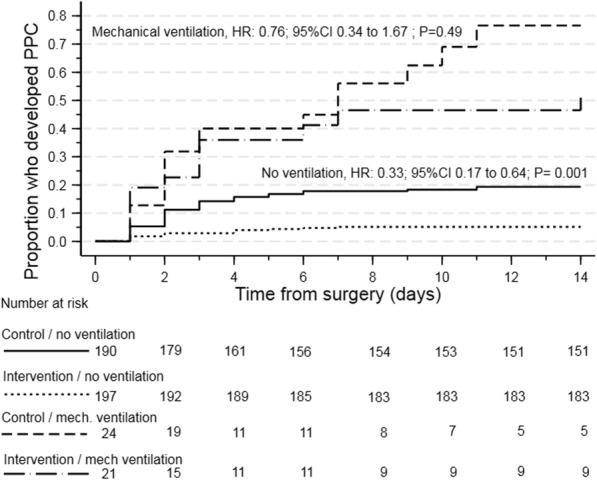
Time to PPC diagnosis sub-grouped to ventilation status immediately after surgery. **a** Conscious and extubated. **b** Sedated and mechanically ventilated. Data are proportions

A large treatment effect in the primary outcome of PPC was found only in the sub-group of participants who were extubated immediately after surgery (RR 0.33, 95% CI 0.17 to 0.64; *p* = 0.001; Fig. 3) with no difference detected in PPC incidence between-groups in participants who remained sedated and mechanically ventilated on completion of surgery.

## Discussion

This exploratory secondary analysis of prospectively collected a priori clinical data within an international, multicentre randomised controlled trial (Boden et al. 2018a) finds that preoperative physiotherapy is associated with the minimisation of clinical signs and symptoms related to postoperative atelectasis and airway infection. Intervention participants were less likely to be prescribed antibiotics specific for a respiratory infection, require oxygen therapy, develop purulent sputum, have positive sputum cultures, or have their episode-of-care coded for a respiratory diagnosis specific to pulmonary collapse or airway infection. Furthermore, the preoperative physiotherapy intervention did not have any effect on complications that have no conceivable physiological basis for breathing exercises to prevent, such as wound infections or pulmonary emboli. Additionally, preoperative physiotherapy was only effective in participants who were extubated and conscious immediately after surgery and therefore more likely to be able to perform self-directed breathing exercises.

These new data provide concurrent validity to the original trial findings of a large significant reduction in PPC incidence following major abdominal surgery in those participants met by a physiotherapist in pre-admission clinics (Boden et al. 2018a). These patients were educated on their risk of a PPC and taught breathing exercises to start performing immediately on waking from surgery. In the absence of direct breathing exercise compliance data, the hypothesis that preoperative physiotherapy engenders the performance of efficacious breathing exercises in the early postoperative period is supported.

Atelectasis is present in almost all patients immediately after major abdominal surgery (Touw et al. 2019; van Kaam et al. 2004; Tusman et al. 2012). Extensive atelectasis causes pulmonary shunt, hypoxemia, and microbial contamination (van Kaam et al. 2004; Tusman et al. 2012). Expert opinion considers that atelectasis is best addressed in the early postoperative period (Ball et al. 2016). At this time, simple lung expansion techniques, such as breathing exercises, may more easily overcome collapsed small airways and the elastic resistance required to re-expand them. If atelectasis progresses to full lobar collapse, breathing exercises may not be effective as greater reductions in lung compliance require significantly more respiratory muscle work to generate the pleural pressure change needed to overcome greater elastic resistance across the lung. The first 24 h may be a vital window where breathing exercises might be most effective (Ball et al. 2016). There is some evidence supporting this theory. Breathing exercises performed immediately postoperatively are reported to reduce atelectasis (Westerdahl et al. 2005) and pulmonary shunt (Ntoumenopoulos and Greenwood 1996), improving lung function (Zoremba et al. 2009), and oxygenation (Manzano et al. 2008), whereas multiple coached breathing exercise sessions initiated on the first postoperative day may not be effective in reducing PPCs (Reeve and Boden 2016; Lunardi et al. 2015). Confirmation that early postoperative breathing exercises can effectively enhance alveolar recruitment is required to further investigate this hypothesis. This could be conducted using point-of-care ultrasound. Ultrasound is more sensitive in detecting atelectasis than CXRs (Ford et al. 2017; Touw et al. 2018), arguably less onerous and harmful to the patient, and could confirm in real-time the proposed physiological and timing of initiation effects of postoperative breathing exercises (Song et al. 2017; Monastesse et al. 2017).

The aim of preoperative physiotherapy is to enable a patient to start performing breathing exercises immediately after surgery, rather than the day after surgery, which is normally when the first physiotherapy session provided (Reeve et al. 2019; van Beijsterveld et al. 2019). Bringing the time point of these exercises forward a day could also introduce a possible dose-dependent relationship benefit. An additional 200 repetitions (20 repetitions, hourly, for 10 h) of deep breathing and coughing exercises are possible if initiated immediately on waking from surgery compared to starting the next day. Preliminary reports find that increased repetitions of breathing exercises augmented with either a positive expiratory pressure device (Urell et al. 2011), or an incentive spirometer with an electronic hourly reminder (Eltorai et al. 2019), significantly improve oxygenation (Urell et al. 2011) and reduce atelectasis (Eltorai et al. 2019) following open cardiac surgery.

Atelectasis traps bronchial secretions creating an environment conducive for microbial contamination (van Kaam et al. 2004; Tusman et al. 2012). In this study, purulent sputum and positive sputum cultures occurred significantly more often in control participants, starting from the second postoperative day. Respiratory infection symptoms take between 24 and 48 h to manifest (Ottosen and Evans 2014). This suggests that the pathogenesis of these increased rates of purulent infected sputum evident on the second postoperative day may have originated in the immediate postoperative period. Breathing exercises performed by intervention participants may have reversed atelectasis in this period thus reducing the risk of airway infection by the second day. It is not surprising that control participants were twice as likely to be prescribed antibiotics specific for a respiratory infection. Sputum colour is the most common reason to trigger an antibiotic prescription by doctors (Butler et al. 2011). Prescribing antibiotics purely on suspicion of hospital-acquired pneumonia appears routine practice (Torres et al. 2010) and contrary to guidelines advising that antibiotics in ward-based patients should only be prescribed on empirical evidence, such as infection on sputum culture (American Thoracic Society 2005). That preoperative physiotherapy independently reduced not only the onset of purulent sputum and positive sputum cultures, but also reduced antibiotic prescriptions has significant implications in assisting efforts to limit antibiotic over-prescription and combating the development of antibiotic resistant bacteria (Guitor and Wright 2018).

No difference between groups was observed for hypoxemia, pyrexia, leukocytosis, auscultation changes, or CXR changes; however, given the inherent reduction in statistical power for secondary outcomes, the lack of observed effects in criteria with low event rates should not be unexpected. Furthermore, these individual criteria are not strongly associated with clinically relevant PPCs. Although common after abdominal surgery, episodic hypoxemia does not appear to be associated with clinically relevant complications rates (Bojesen et al. 2019); leukocytosis is a general sign of an immune response and lacks specificity to pulmonary infection alone (Alazawi et al. 2016); auscultation has poor sensitivity and reliability as a stand-alone measure of respiratory dysfunction (Xavier et al. 2019); and the association between fever and atelectasis is hotly contested (Crompton et al. 2019). As discussed earlier, preliminary research is finding that ultrasound, rather than CXR or CT, could be a more sensitive measure of respiratory dysfunction after surgery (Ford et al. 2017; Touw et al. 2018; Song et al. 2017; Monastesse et al. 2017).

Hospitals routinely collect clinical coding for billing and epidemiology purposes. For this trial, clinical coding outcomes add concurrent validity to primary results. The increased documentation in the medical record of respiratory complications specific to pulmonary collapse and airway infection in control participants were clinically significant enough to be detected by masked coders analysing only the medical record. A difference between groups was only detected in respiratory complications considered responsive to breathing exercises, and not for pneumothorax, pulmonary emboli, and pleural effusions, and all other general intra-operative and non-respiratory postoperative complications with no conceivable physiological basis for breathing exercises to effect. These data should be considered with caution, however, as coding under-report true event rates and lack reliability (Koch et al. 2012).

Early self-directed breathing exercises most likely minimise signs and symptoms of atelectasis and pulmonary infection after surgery but only if patients remember the exercises and perform them as instructed. The memorability of this intervention has been previously demonstrated with 94% of intervention participants recalling the breathing exercises taught compared to 15% who received the instructions in written form only (Boden et al. 2018b). Furthermore, these secondary analyses demonstrate that no benefit was achieved from having received preoperative physiotherapy in patients who remained mechanically ventilated and sedated after surgery. This is likely due to their inability to perform the breathing exercises as taught.

In LIPPSMAck-POP, the only prophylactic respiratory physiotherapy participants received was a single session of preoperative education and breathing exercise training. This simple intervention reduced PPCs by half (RR 0.48, 95% CI 0.30 to 0.75) (Boden et al. 2018a), a superior result to multiple sessions of postoperative coached breathing exercises (Reeve and Boden 2016; Lunardi et al. 2015) and incentive spirometer use (do Nascimento Junior et al. 2014). It also delivered comparative PPC reduction benefits to modern perioperative surgical and anaesthetic practices such as lung protective ventilation, prophylactic non-invasive ventilation, goal-directed fluid therapy, and epidural analgesia (Odor et al. 2020).

Although systematic reviews find that physiotherapy is an effective modality to reduce the risk of PPCs the most efficacious and cost-effective physiotherapy technique is unknown (Odor et al. 2020; Hanekom et al. 2012). Most physiotherapy trials were conducted more than 10 years ago, were of low quality, tested multimodal interventions, and did not adequately control or measure known confounders, such as early ambulation and other perioperative practices aimed at PPC minimisation. LIPPSMAck-POP rigorously demonstrated that a single preoperative physiotherapy session conducted in the context of standardised early ambulation, enhanced recovery pathways, and modern perioperative anaesthetic and surgical practices independently minimises PPCs without the use of additional postoperative coached breathing exercises or devices and is cost-effective (Boden et al. 2020). Trials are now required to determine if the addition of other interventions such as inspiratory muscle training, prehabilitation, or, postoperative coached breathing exercises and non-invasive ventilation confer any additional clinical benefit or are cost-effective compared to providing preoperative physiotherapy alone.

## Conclusion

These secondary analyses contribute to original primary findings that a single preoperative session with a physiotherapist halves the risk of a PPC after major abdominal surgery. This paper reports associated clinical benefits to signs of airway infection, oxygen therapy use, and antibiotic prescription rates. In developed countries, preoperative physiotherapy is currently not standard practice (Reeve et al. 2019; van Beijsterveld et al. 2019). This new evidence, in conjunction with cost-effectiveness (Boden et al. 2020) and consumer-lead preference for this service (Boden et al. 2018b), may now encourage hospitals to consider embedding a preoperative physiotherapy service within pre-admission clinics for all patients listed for elective upper abdominal surgery.

## Supplementary Information

**Additional file 1: Box 1S.** Postoperative pulmonary complication diagnostic criteria. **Table 1S** Methodological instructions for collection of clinical criteria within the Melbourne Group Score postoperative pulmonary complication diagnostic tool. **Table 2S**. List of International Classification of Diseases (ICD-10) clinical codes extracted. **Figure 1S** Proportion of participants with positive clinical criteria on each postoperative day.

## Data Availability

The datasets used and analysed during the current study are available from the corresponding author on reasonable request. The full LIPPSMAck-POP dataset is currently being produced for planned open-access publication alongside the primary publication here: https://www.bmj.com/content/360/bmj.j5916.
