# Supplementary material for: Effects of preoperative physiotherapy on signs and symptoms of pulmonary collapse and infection after major abdominal surgery: secondary analysis of the LIPPSMAck-POP multicentre randomised controlled trial
Source: Perioper Med (Lond). 2021 Oct 25;10:36. doi: 10.1186/s13741-021-00206-3 (PMC8543902; doi:10.1186/s13741-021-00206-3)

Additional File 1

Box 1S Postoperative pulmonary complication diagnostic criteria.

Table 1S Methodological instructions for collection of clinical criteria within the Melbourne Group Score postoperative pulmonary complication diagnostic tool.

Table 2S List of International Classification of Diseases (ICD-10) clinical codes extracted

Figure 1S Proportion of participants with positive clinical criteria on each postoperative day.

Box 1S: Postoperative pulmonary complication diagnostic criteria.

Diagnosis confirmed when **4 or more** of the following were present in a calendar day:

CLINICAL CRITERIA

1. New abnormal breath sounds on auscultation different to pre-operative assessment
2. Production of yellow or green sputum different to pre-operative assessment
3. SpO_2 <90% on room air, two episodes over two consecutive postoperative day
4. Maximum temperature >38°C, two episodes over two consecutive postoperative day

DIAGNOSTIC CRITERIA

5. CXR/CT report of collapse/consolidation
6. An unexplained WCC greater than 11×10^9/l
7. Presence of infection on sputum culture report

CLINICIAN RESPONSE CRITERION; either of below

8. a) Physician's diagnosis documentation in the medical record of pneumonia, URTI, LRTI, or respiratory problem, **OR,**
   b) Prescription of an antibiotic specific for a respiratory infection

Abbreviations: SpO_2=pulse oximetry oxygen saturation, CXR=chest x-ray, CT=computerised tomography, WCC=white cell count, URTI=upper respiratory tract infection, LRTI=lower respiratory tract infection

Table 1S: Methodological instructions for collection of clinical criteria within the Melbourne Group Score postoperative pulmonary complication diagnostic tool.

Auscultation	Sit patient in upright position if possible. Auscultate all zones of the lungs, apical, basal, anterior, and posterior.<br>Record any of the following findings:<br>- Reduced air entry<br>- Bronchial breath sounds or rales<br>- Crackles or creps<br>- Any added sounds that are abnormal<br>If any of these sounds are worse than the preoperative record, record as positive.
Sputum change	Review medical record for any record of coloured sputum being coughed or suctioned since time of last assessment.<br>Question participant about any production of coloured sputum since last assessment and, if possible, visualise a sample.<br>If the participant is mechanically ventilated interrogate the colour of the last suctioned secretions.<br>Compared colour to documentation of preoperative findings using the standardised colour chart provided.<br><br>Any new yellow-green-brown sputum or change of colour from preoperative reports in each postoperative 24hrs (midnight to midnight), documented in the medical record or measured directly, is recorded as positive.
SpO_2 on room air	Review medical record for any desaturation events since time of last assessment. If medical record of desaturation in the time from last assessment to midnight of that day, record as a positive for that postoperative day.<br>For each daily assessment, sit patient in upright position if possible.<br>Remove supplemental oxygen, or in ventilated patients reduce fraction of inspired oxygen (FiO_2) to 0.21.<br>Measure pulse oximetry oxygen saturation (SpO_2) continuously for two minutes.<br>If SpO_2 became less than 90% immediately reapply oxygen and monitor patient for another two minutes or until back to baseline SpO_2. If SpO_2 remains below baseline, contact site investigator and record as an adverse measurement event. Continue monitoring for a further 2 minutes. If remains <92% after this time contact Senior Nurse.<br><br>Any desaturation event in the 24hrs (midnight to midnight) either documented in the medical record or measured directly is recorded as positive.
Temperature	Review observation chart and medical record for any febrile events >38C since time of last assessment. If medical record of pyrexia in the time from last assessment to midnight of that day, record as a positive for that postoperative day.

Table 2S: List of International Classification of Diseases (ICD-10) clinical codes extracted

Code		Description
A41.9		Sepsis, unspecified
E86		Volume depletion
E87.7		Fluid overload
E87.0		Hyperosmolality and hypernatremia
E87.6		Hypokalemia
E87.7		Fluid overload
F05.9		Delirium, unspecified
I211.1		Acute transmural mycocardial infarction of inferior wall
I26.0 – I26.9	+	Pulmonary embolism
I440		AV block
I46.0		Cardiac arrest with successful resusitation
I48.9		Atrial fibrillation and flutter, unspecified
I82.8		Embolism and thrombosis of other specified veins
J00 – J06	*	Acute upper respiratory infections, including pharyngitis, sinusitis, rhinitis, tonsillitis
J06	*	Acute upper respiratory infections of multiple and unspecified sites
J06.9	*	Acute upper respiratory infection, unspecified
J09 – J18	*	Influenza and pneumonia
J20 – J22	*	Other acute lower respiratory infections
J22	*	Unspecified acute lower respiratory infection
J30 – J39	*	Other diseases of upper respiratory tract including rhinitis, pharyngitis, nasal polyps,
J40 – J47		Chronic lower respiratory diseases
J44		Other chronic obstructive pulmonary disease
J44.0	*	Chronic obstructive pulmonary disease with acute lower respiratory infection
J44.1	*	Chronic obstructive pulmonary disease with acute exacerbation, unspecified
J60 – J70	+	Lung diseases due to external agents
J69.0	+	Pneumonitis due to food and vomit
J80 – J84	+	Other respiratory diseases principally affecting the interstitium
J80	+	Adult respiratory distress syndrome
J81	+	Pulmonary oedema
J84.0 – J84.9	+	Other interstitial pulmonary diseases
J90 – J94	+	Other diseases of pleura
J90	+	Pleural effusion, not elsewhere classified
J93 – J93.8	+	Pneumothorax
J94	+	Other pleural conditions
J95		Post procedural respiratory disorders, not elsewhere classified
J95.2	+	Acute pulmonary insufficiency following nonthoracic surgery
J95.8	+	Other post procedural respiratory disorders
J95.9	+	Postprocedural respiratory disorder, unspecified
J96	*	Respiratory failure, not elsewhere classified
J96.0	*	Acute respiratory failure, type I
J96.1	*	Acute respiratory failure, type II
J96.9	*	Respiratory failure unspecified
J96.90	*	Respiratory failure unspecified, type I
J96.09	*	Acute respiratory failure, type unspecified
J96.99	*	Respiratory failure unspecified, type unspecified
J98		Other respiratory disorders

J98.0	*	Diseases of bronchus, not elsewhere classified
J98.1	*	Pulmonary collapse
J98.8	*	Other specified respiratory disorders
J99	+	Respiratory disorders in diseases classified elsewhere<br>Includes rheumatoid lung disease and connective tissue disorders related lung disease
K56.7		Ileus, unspecified
K59.0		Constipation
K91.3		Postprocedural intestinal obstruction
L23.8		Decubitus ulcer and pressure area, unspecified
L89.1		Stage II decubitus ulcer and pressure area
N17.9		Acute kidney failure
N39.0		Urinary tract infection, site not specified
R00.0 – R00.8		Abnormalities of heart beat
R05	+	Cough
R06	+	Abnormalities of breathing
R06.0	+	Dyspnoea
R06.8	+	Other and unspecified abnormalities of breathing
R09.3	*	Abnormal sputum
R09.89		Other specified symptoms and signs involving the respiratory system
R40-R46		Symptoms and signs involving cognition, perception, emotional state, and behaviour
R40.0 – R40.2		Somnolence, stupor, and coma
R41.0		Disorientation, unspecified
R44.0 – R44.8		Other symptoms and signs involving cognitive functions and awareness
R45.1		Restlessness and agitation
R44		Other symptoms and signs involving general sensations and perceptions including hallucinations
R53		Malaise and fatigue
R55		Syncope and collapse
R58		Haemorrhage, not elsewhere classified
R65.0 – R65.9		Systemic inflammatory response syndrome
T81.0		Haemorrhage and haematoma complicating a procedure, not elsewhere classified
T81.2		Accidental puncture and laceration during a procedure, not elsewhere classified
T81.3		Disruption of operation wound, not elsewhere classified
T81.4		Infection following a procedure, not elsewhere classified
T81.41		Wound infection following a procedure
T857.8		Infection and inflammatory reaction due to other internal prosthetic devices, implants, and grafts
Other infection		Cystitis N30.9, Candidiasis of skin and nail B37.2, cellulitis of upper limb L031.0
Wound infection		Candidal stomatitis B37.0

*codes classified as respiratory diagnoses related to pulmonary collapse or airway infection
+ codes classified as respiratory diagnoses not directly related to pulmonary collapse or airway infection

Figure 1S: Proportion of all trial participants with positive clinical criteria on each postoperative day.

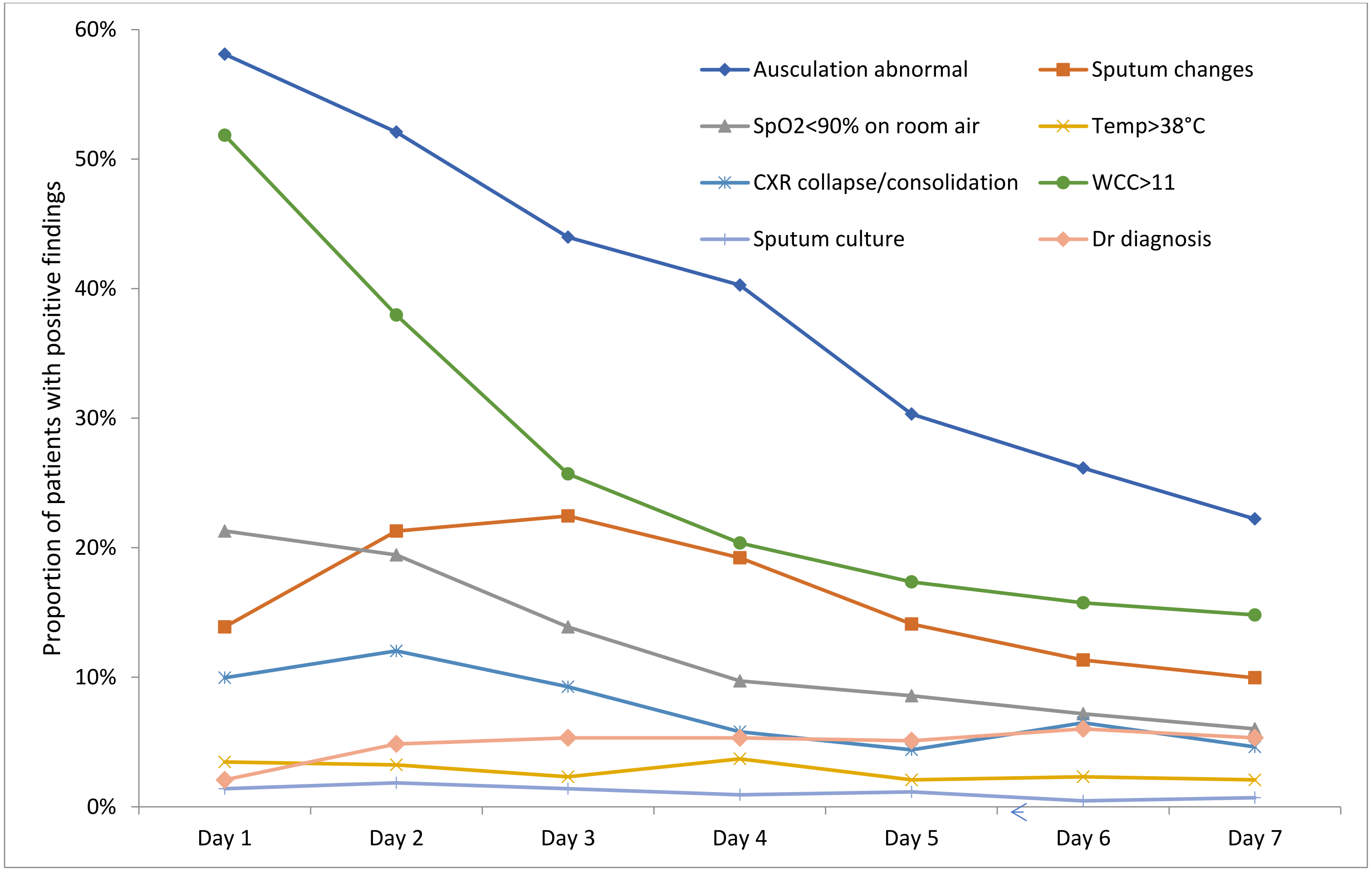

Supplement: Supplementary file 1 — Additional file 1: Box 1S. Postoperative pulmonary complication diagnostic criteria. Table 1S Methodological instructions for collection of clinical criteria within the Melbourne Group Score postoperative pulmonary complication diagnostic tool. Table 2S. List of International Classification of Diseases (ICD-10) clinical codes extracted. Figure 1S Proportion of participants with positive clinical criteria on each postoperative day. [file 13741_2021_206_MOESM1_ESM.pdf]
